# Top and bottom longevity of nations: a retrospective analysis of the age-at-death distribution across 18 OECD countries

**DOI:** 10.1093/eurpub/ckac134

**Published:** 2022-10-14

**Authors:** Stéphane Verguet, Miyu Niwa, Sarah Bolongaita

**Affiliations:** Department of Global Health and Population, Harvard T.H. Chan School of Public Health, Boston, MA, USA; Department of Global Health and Population, Harvard T.H. Chan School of Public Health, Boston, MA, USA; Department of Global Health and Population, Harvard T.H. Chan School of Public Health, Boston, MA, USA

## Abstract

**Background:**

Similar to the study of the distribution of income within countries, population-level health disparities can be examined by analyzing the distribution of age at death.

**Methods:**

We sourced period-specific death counts for 18 OECD countries over 1900–2020 from the Human Mortality Database. We studied the evolution of country-year-specific distributions of age at death, with an examination of the lower and upper tails of these distributions. For each country-year, we extracted the 1st, 5th, 10th, 90th, 95th and 99th percentiles of the age-at-death distribution. We then computed the corresponding shares of longevity—the sum of the ages weighted by the age-at-death distribution as a fraction of the sum of the ages weighted by the distribution—for each percentile. For example, for the 10th percentile, this would correspond to how much longevity accrues to the bottom 10% of the age-at-death distribution in a given country-year.

**Results:**

We expose a characterization of the age-at-death distribution across populations with a focus on the lower and upper tails of the distribution. Our metrics, specifically the gap measures in age and share across the 10th and 90th percentiles of the distribution, enable a systematic comparison of national performances, which yields information supplementary to the cross-country differences commonly pointed by traditional indicators of life expectancy and coefficient of variation.

**Conclusions:**

Examining the tails of age-at-death distributions can help characterize the comparative situations of the better- and worse-off individuals across nations, similarly to depictions of income distributions in economics.

## Introduction

Improving the distribution of population health is a major goal of national health systems.^1^^,^^2^ Health sector policies can be envisioned as redistribution instruments to redress inequalities in societies, with the specific intent of narrowing the gaps between the better-off (e.g. the wealthiest) and the worse-off (e.g. the poorest) citizens. In this respect, quantifying the extent of the health gaps between ‘extreme’ groups in societies may be helpful to characterize some of the health disparities prevailing in a given country. One possibility to do so is to study the distribution of the age at death in populations.

Seminal works have documented and analyzed the distribution across socioeconomic groups (e.g. income and wealth quintiles) of health summary measures including age-specific mortality rates, life expectancy and disease-specific outcomes; they have successfully pointed to the great significance of the social determinants of health.^3^^,^^4^ As a complement, other scholars have paid attention to the full distribution of health in populations, without disaggregating health outcomes across population subgroups, much like economists have been routinely examining the distribution of income in populations.^5–7^ Importantly, a large literature in demography has long studied the distributions of mortality outcomes with detailed analyses of the distributions of age at death. An abundant scholarship developed and characterized disparities in lifespan, with the use of various summary indicators like interquartile ranges, Gini indices or rectangularities of survival curves.^8–16^

Most recently, there has been substantial attention to the widening of income inequalities and to the ever-increasing gaps between bottom and top income earners within countries, especially in high-income countries,^17–21^ with the richest of the income distribution expanding their wealth at fast rates compared with the poorest of that distribution. This prompts to the design of enhanced redistribution policies and to potentially rethink the design of fiscal policies. Inspired by this economic literature, we intend to apply a similar lens to the examination of the full distribution of health (in place of income or wealth) across countries. Specifically, we aim to study comparatively across countries the evolution of the lower and upper tails of the distribution of age at death. Using high-quality data for 18 sufficiently large countries over 1900–2020 from the Human Mortality Database (HMD),^22^ the objective is to extract easy-to-use summary quantities, comparable to those from the income inequality literature, that could capture the concentration of top and bottom health (proxied by longevity here) in societies.

## Methods

We start by describing the mortality data sources used, and then detail the analysis and sets of computations conducted with the data.

### Data sources

We extracted period-specific numbers of deaths from 18 countries over 1900–2020 from the HMD (HMD 2021) (Supplementary table SA1). We restricted the selection of countries to those countries with population >1 million inhabitants as of 2020^23^ and to those countries belonging to the Organization for Economic Co-operation and Development (OECD).^24^ For all countries and years, the number of deaths was available by single-year age group from age 0–110, disaggregated by sex.

### Analytical approach

Using the HMD data, for a given country c and year t, we denoted: n(x,c,t), the number of deaths at age x; and d(x,c,t), the distribution of age at death. By definition, d(x,c,t) (denoted d(x) thereafter for simplicity) is given by $d\left(x\right)=\frac{n\left(x\right)}{\int_{0}^{\omega }n\left(u\right)du}$, where ω is the highest age group reported in the population (here ω = 110). (By construction, for any given country-year, we have: $\int_{0}^{\omega }d\left(x\right)dx=1$.)

We studied the percentile [e.g. 1st (0.01), 5th (0.05), 10th (0.10), 90th (0.90), 95th (0.95) and 99th (0.99)] of this age-at-death distribution (for any country-year) by analyzing the age $a_{p}$ that is extracted from the designated percentile (p = {0.01; 0.05; 0.10; 0.90; 0.95; 0.99}) in the following way:$\;\int_{0}^{a_{p}}d\left(x\right)dx=p$. We could then derive: $a_{0.01}$, $a_{0.05}$, $a_{0.10}$, $a_{0.90}$, $a_{0.95}$ and $a_{0.99}$.

Subsequently, we computed the share of ‘health’ (here proxied by a fraction of longevity in the country-year population) that corresponded to each percentile p: $F_{p-}=\frac{\int_{0}^{a_{p}}xd\left(x\right)dx}{\int_{0}^{\omega }xd\left(x\right)dx}$ when 0<p≤0.10, for the bottom tail of the distribution; and $F_{p+}=\frac{\int_{a_{p}}^{\omega }xd\left(x\right)dx}{\int_{0}^{\omega }xd\left(x\right)dx}$ when 0.90≤p<1, for the top tail of the distribution. $F_{p-}$ intends to quantify the share among the bottom percentiles of the distribution of age at death (for p = {0.01; 0.05; 0.10}); while$\;F_{p+}$ intends to quantify the share among the top percentiles of the distribution (for p= {0.90; 0.95; 0.99}).

We could then compare the different percentile-derived quantities among themselves by studying across countries and years the following summary ‘distance’ (gap) indicators: such as age gaps with$\;\delta_{0.01}=a_{0.99}-a_{0.01}$, $\delta_{0.05}=a_{0.95}-a_{0.05}$, $\delta_{0.10}=a_{0.90}-a_{0.10}$; and, share gaps with $\Delta_{0.01}=F_{0.99}-F_{0.01}$, $\Delta_{0.05}=F_{0.95}-F_{0.05}$, $\Delta_{0.10}=F_{0.90}-F_{0.10}$. This provides one easy-to-use approach to compare countries over time.

All calculations were conducted with R software (www.r-project.org).

## Results

**Figure 1:**
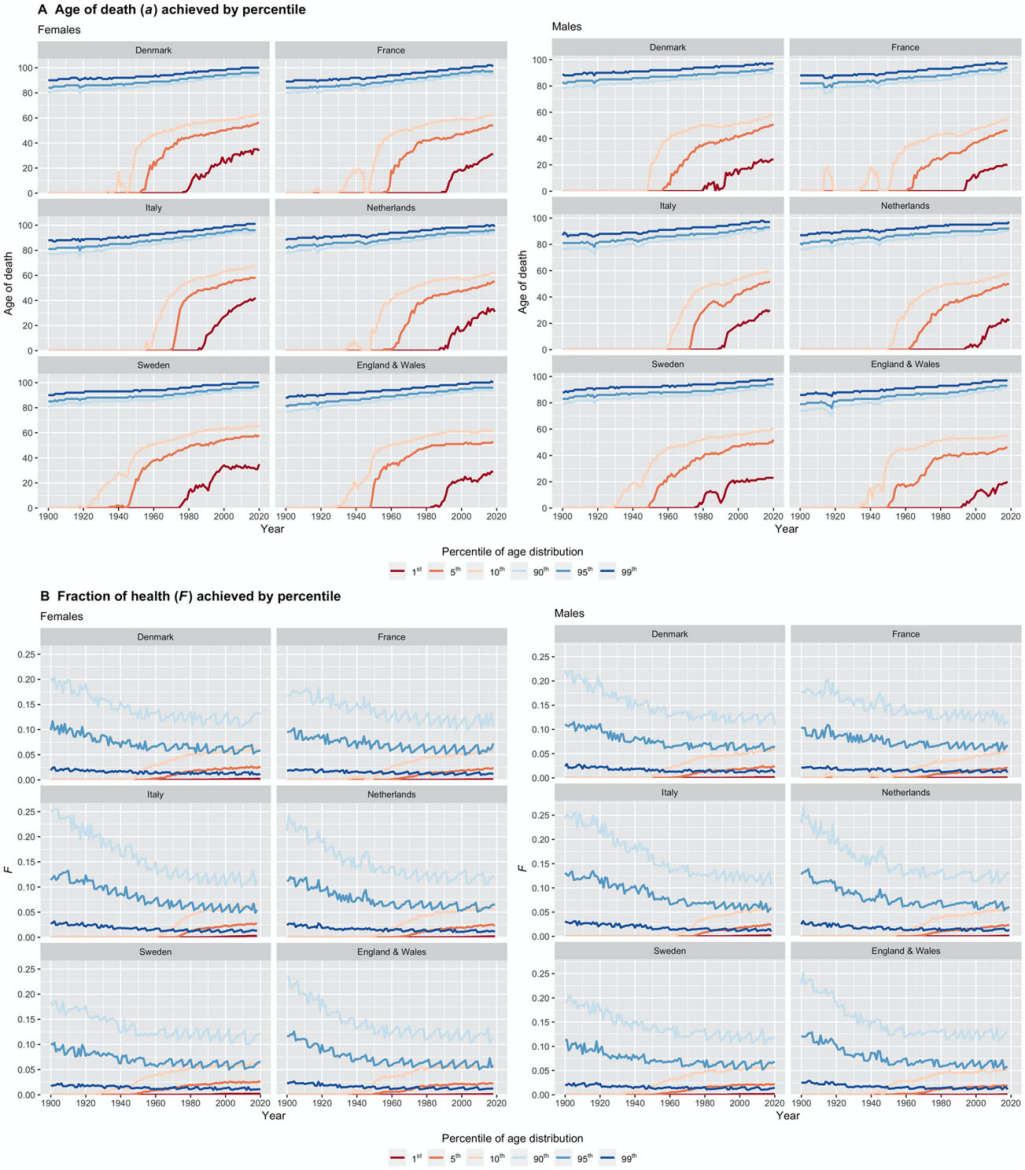
(A) Age at death (in years) achieved by the following percentiles of the age-at-death distribution: $a_{0.01}$, $a_{0.05}$, $a_{0.10}$, $a_{0.90}$, $a_{0.95}$, and $a_{0.99}$. (B) Fraction of health (longevity) achieved by the following age-at-death percentiles: $F_{0.01}$, $F_{0.05}$, $F_{0.10}$, $F_{0.90}$, $F_{0.95}$ and $F_{0.99}$, Denmark, England and Wales, France, Italy, the Netherlands and Sweden; females and males; 1900–2020

We first report on the evolution over time of the age at death at selected percentiles of the distribution ($a_{p}$) for six countries (or areas) with sufficiently long time periods of data (>100 years) and large enough populations: Denmark, England and Wales, France, Italy, the Netherlands and Sweden (figure 1A). Across all countries, for both females and males, we see that the top percentiles (i.e. $a_{0.90}$, $a_{0.95}$ and $a_{0.99}$) remained relatively steady, with slightly increasing high ages at death over time (roughly within 80–100 years). On the other hand, the age at death for the bottom percentiles ($a_{0.10}$, $a_{0.05}$ and $a_{0.01}$) evolved more abruptly over time, with rapid shifts upwards: from 0 toward around 60–70 years after around 1940–60 for $a_{0.10}$, from 0 toward around 40–60 years after around 1950–70 for $a_{0.05}$ and from 0 toward around 20–40 years after around 1980–2000 for $a_{0.01}$. In between, $a_{0.50}$ (not shown) increased substantially, due to large reductions in mortality within the younger age groups (i.e. the bottom half of the age-at-death distribution, linked notably to the evolutions of $a_{0.10}$, $a_{0.05}$ and $a_{0.01}$). These patterns are confirmed when analyzing the corresponding ‘distance’ (age gap) quantities $\delta_{0.10}$, $\delta_{0.05}$ and $\delta_{0.01}$ (Supplementary figure SA1): we observe important decreases across countries, for both females and males, especially so for $\delta_{0.10}$ and $\delta_{0.05}$, which points to a thinning of the age-at-death distribution over time.

**Figure 2:**
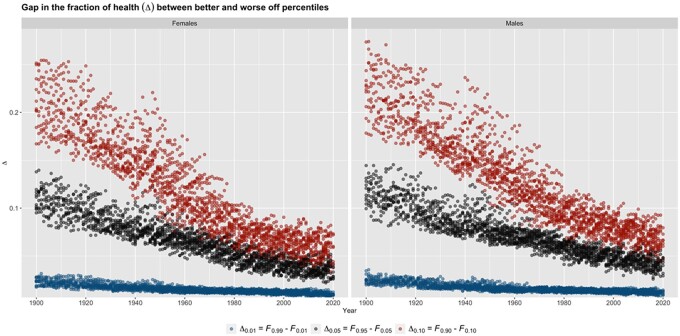
Gap in the fraction of health (longevity) between top and bottom percentiles of age at death: $\Delta_{0.10}=F_{0.90}-F_{0.10}$, $\Delta_{0.05}=F_{0.95}-F_{0.05}$ and $\Delta_{0.01}=F_{0.99}-F_{0.01}$; all country-years; females and males; 1900–2020

Second, we describe the evolution over time of the shares of longevity ($F_{p}$) for the same six countries (or nations) (figure 1B). Across all countries, for both females and males, we observe that the share captured by the top percentiles ($F_{0.90}$, $F_{0.95}$ and $F_{0.99}$) has decreased over time, while that of the bottom percentiles ($F_{0.10}$, $F_{0.05}$ and $F_{0.01}$) has increased, which points to longevity gaps narrowing over time. Steady reductions in F among the top percentiles can be seen throughout the entire time period: roughly, from around 18–25% to around 10–13% for $F_{0.90}$, and from around 10–13% to around 5–7% for $F_{0.95}$. Among the bottom percentiles, shifts upwards occur noticeably later, spanning the 1930s through the 1970s: toward around 5–7% for $F_{0.10}$, and toward around 2–3% for $F_{0.05}$. (The fractions $F_{0.01}$ appear too small to enable the reporting of interpretable estimates.) Across country-years, we see that the share gaps across the top and bottom percentiles narrow over time (figure 2 and Supplementary figure SA2). This is particularly true for $\Delta_{0.10}$ (from as high as around 30% to as low as around 5%, with an apparent deceleration in declines over around the 1970–1980s) and to a lesser extent for $\Delta_{0.05}$ (from as high as about 15% to as low as about 3%, with somewhat more linear decreases over time). [The variations in $\Delta_{0.01}$ are much smaller (within 2%), as this is largely due to the small quantities computed (either small ages or small shares), especially so for $F_{0.01}$.]

**Figure 3:**
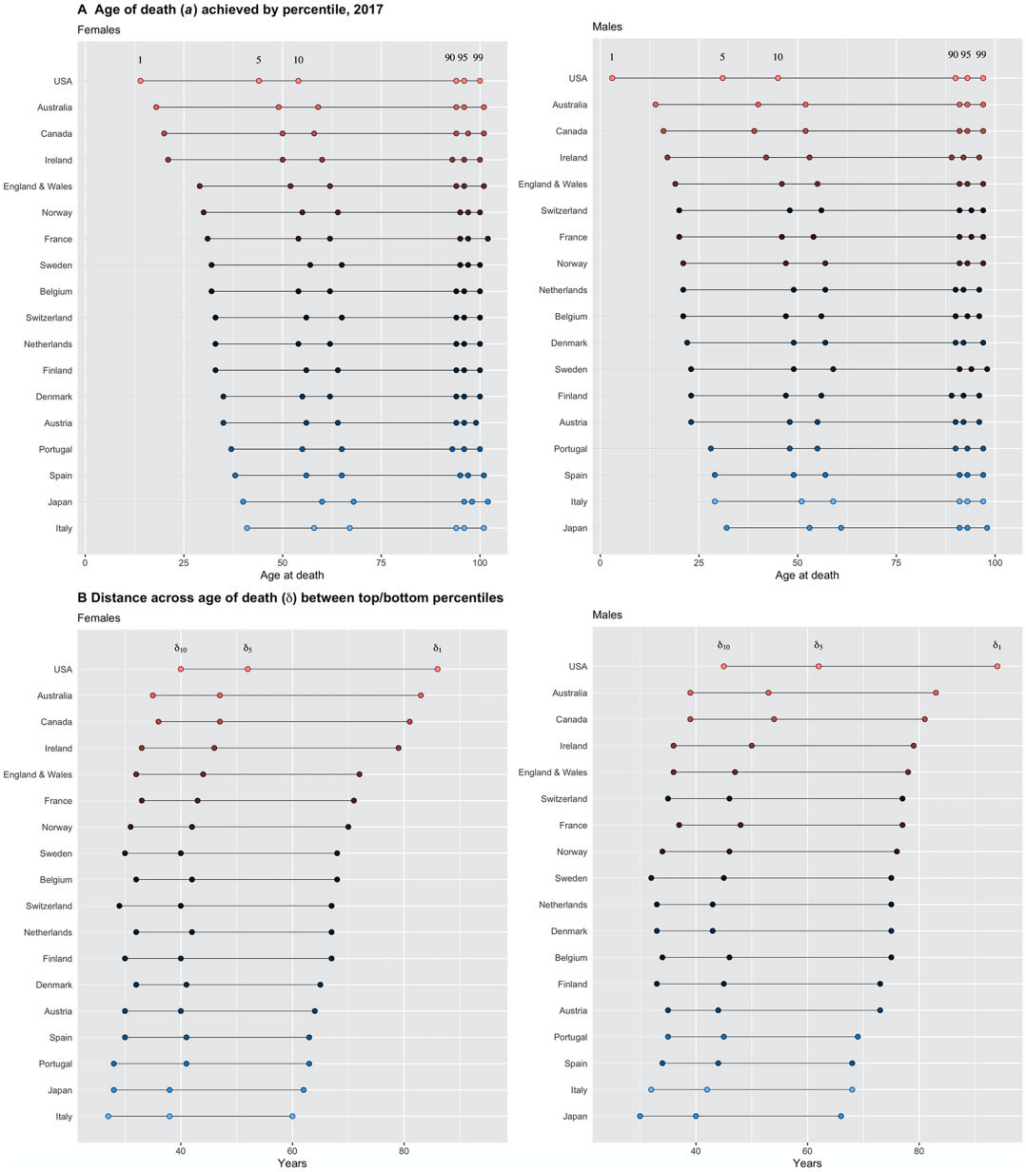
(A) Age at death (in years) achieved by the different percentiles of the 2017 age-at-death distribution: $a_{0.01}$ (1), $a_{0.05}$ (5), $a_{0.10}$ (10), $a_{0.90}$ (90), $a_{0.95}$ (95) and $a_{0.99}$ (99). (B) Distance (in years) across age at death between top and bottom percentiles: ${\delta_{0.10}=a}_{0.90}-a_{0.10}\;(10)$, ${\delta_{0.05}=a}_{0.95}-a_{0.05}\;(5)$ and ${\delta_{0.01}=a}_{0.99}-a_{0.01}\;(1)$; 18 countries, females and males

Third, some of these quantities could be used to compare the performance in those age-at-death distributions across countries, e.g. by pointing to the differences observed in the age at death corresponding to the selected percentiles (see figure 3A for a display for the year 2017, the last year for which data were available for all 18 countries in the study sample). For both females and males, we see little variations in $a_{0.90}$, $a_{0.95}$ and $a_{0.99}$ across countries, roughly of a few years spanning over 90–100 years, consistent with what we highlighted previously (figure 1). However, we see much larger variations across countries in $a_{0.10}$ and $a_{0.05}$ (of around 10–20 years), and especially so for $a_{0.01}$ (of as high as 25 years or so). As an illustration, $a_{0.01}$ could range from below 5 years for US males to around 40 years for Italian females. Similarly, we see large heterogeneities across countries in terms of when (i.e. which calendar year) $a_{0.10}$, $a_{0.05}$ and $a_{0.01}$ became >0 (Supplementary figure SA3).

As a result, the ‘distance’ (age gap) indicators $\delta_{0.10}$, $\delta_{0.05}$ and $\delta_{0.01}$ largely depend on $a_{0.10}$, $a_{0.05}$ and $a_{0.01}$ (figure 3B, with 2017 estimates). On the one hand, we see that the USA is among the poorest performers for both females and males, with $\delta_{0.01}$ > 85, $\delta_{0.05}$ > 50 and $\delta_{0.10}$ > 40 (years). On the other hand, we see that Italian females (closely followed by Japanese females) would do well with $\delta_{0.01}\;∼\;$60, $\delta_{0.05}$ < 40 and $\delta_{0.10}$ < 30 (years). Also, Japanese males (closely followed by Italian males) would do well with $\delta_{0.01}$ < 70, $\delta_{0.05}\;∼$ 40 and $\delta_{0.10}\;∼$ 30 (years).

When observing the estimated shares of longevity (F, for the post-2000 years), expectedly, we find little amplitude across country-years for $F_{0.01}$ and $F_{0.99}$ (within 0–2%), which indicates that the shares achieved by the bottom and top one percentiles of the age-at-death distribution are not easily quantifiable thus interpretable across countries in recent years (Supplementary figure SA4). Meanwhile, the greatest amplitude and variation are observed for the 90th percentile ($F_{0.90}$). On average, males (compared to females) appear to have larger F shares within the top tail (90th, 95th and 99th percentiles) as well as lower F shares within the bottom tail (10th, 5th and 1st percentiles) (Supplementary figure SA4). Males also have larger share gaps (Δ’s), which indicates greater disparities in the shares of longevity obtained along the male age-at-death distribution (Supplementary figure SA5).

We now turn to $\Delta_{0.10}$, the share gap indicator that presented the largest amplitude (hence stability, compared with $\Delta_{0.05}$ and $\Delta_{0.01}$). For females, the poorest performances were for the USA and France, with 7.7% and 7.5% share gaps, respectively, for the year 2017; while the best performances were for Italy and Japan, with 3.9% and 5.2%, respectively, for 2017. As for males, the poorest performances were for the USA and Australia, with 9.4% and 8.9%, respectively; while the best performances were for Portugal and Switzerland, with 5.1% and 5.4%, respectively (table 1). Furthermore, we report on the correlation between $\Delta_{0.10}$ and life expectancy at birth: correlation of −0.42 (*P* = 0.09) for females and of −0.34 for males (*P* = 0.17) [with rank correlations of 0.41 (*P* = 0.09) and 0.55 (*P* = 0.02), respectively]. When comparing with the coefficient of variation (of the age-at-death distributions), we observe: correlation of 0.48 (*P* = 0.05) for females and 0.55 (*P* = 0.02) for males [with rank correlations of 0.48 (*P* = 0.04) for females and 0.16 (*P* = 0.52), respectively]. We also computed the same correlations for a 30-year period (1987–2017; *n* = 1550 data points). $\Delta_{0.10}$ and life expectancy then exhibited a correlation of −0.43 (*P* < 0.001) for females and of −0.54 for males (*P* < 0.001) [for ranks: −0.37 (*P* < 0.001) and −0.55 (*P* < 0.001), respectively]. $\Delta_{0.10}$ and coefficient of variation then showed a correlation of 0.59 (*P* < 0.001) for females and of 0.56 for males (*P* < 0.001) [for ranks: 0.25 (*P* < 0.001) and 0.39 (*P* < 0.001), respectively]. Such ranges of partial correlation point to what extent the share gap indicators (here $\Delta_{0.10}$) could serve to highlight distinct features of disparities between ‘extreme’ groups within countries (via the bottom and top tails of age-at-death distributions), and supplement the fuller pictures of disparities in longevity (as coefficients of variation or Gini indices do).

**Table 1:** Gap in the share of longevity between top and bottom percentiles (in %): $\Delta_{0.01}=F_{0.99}-F_{0.01}$, $\Delta_{0.05}=F_{0.95}-F_{0.05}$ and $\Delta_{0.10}=F_{0.90}-F_{0.10}$; 18 countries; females and males; 2017

Females							
Rank	**Country—** $\Delta_{0.01\;}$ **(%)**	**Country—** $\Delta_{0.05}\;$ **(%)**	**Country—** $\Delta_{0.10}\;$ **(%)**	*F*_0.10_ (%)	*F*_0.90_ (%)	Life expectancy (years) [rank]	Coefficient of variation [rank]
18	Canada 1.45	Canada 4.64	USA 7.68	5.02	12.69	81.36 [18]	0.52 [18]
17	Australia 1.23	France 4.07	France 7.54	5.68	13.22	85.36 [3]	0.39 [13]
16	Ireland 1.21	Ireland 4.00	Austria 7.06	5.74	12.79	83.90 [12]	0.36 [5]
15	USA 1.20	Australia 3.94	Denmark 7.03	6.20	13.24	83.12 [17]	0.36 [6]
14	England and Wales 1.14	USA 3.85	Canada 6.39	5.47	11.86	84.11 [11]	0.47 [16]
13	Portugal 1.13	The Netherlands 3.82	Norway 6.32	5.80	12.12	84.28 [8]	0.39 [12]
12	France 1.12	Finland 3.79	Belgium 6.20	5.86	12.05	83.65 [14]	0.38 [11]
11	Spain 1.10	Norway 3.61	The Netherlands 6.06	5.86	11.92	83.32 [15]	0.38 [10]
10	The Netherlands 1.09	Portugal 3.60	Australia 5.97	5.35	11.31	84.99 [5]	0.48 [17]
9	Italy 1.08	Spain 3.52	Japan 5.75	6.69	12.44	87.31 [1]	0.33 [2]
8	Belgium 1.06	Austria 3.50	Finland 5.69	6.35	12.05	84.22 [9]	0.37 [8]
7	Finland 1.00	Sweden 3.47	Sweden 5.68	6.09	11.77	84.12 [10]	0.37 [9]
6	Switzerland 0.98	Belgium 3.46	Ireland 5.57	5.44	11.01	83.73 [13]	0.45 [15]
5	Denmark 0.96	England and Wales 3.23	England and Wales 5.50	5.54	11.04	83.20 [16]	0.40 [14]
4	Austria 0.88	Switzerland 3.22	Switzerland 5.34	5.80	11.14	85.36 [3]	0.36 [7]
3	Norway 0.87	Denmark 3.05	Portugal 5.30	5.99	11.29	84.38 [7]	0.35 [4]
2	Sweden 0.84	Japan 2.32	Spain 5.15	6.13	11.28	85.68 [2]	0.35 [3]
1	Japan 0.67	Italy 2.19	Italy 3.92	6.51	10.43	84.86 [6]	0.32 [1]

Males							
Rank	**Country—** $\Delta_{0.01}\;$ **(%)**	**Country—** $\Delta_{0.05}\;$ **(%)**	**Country—** $\Delta_{0.10\;}$ **(%)**	*F*_0.10_ (%)	*F*_0.90_ (%)	Life expectancy (years) [rank]	Coefficient of variation [rank]
18	USA 1.69	Ireland 5.39	USA 9.35	4.06	13.41	76.31 [18]	0.65 [18]
17	England and Wales 1.56	USA 5.39	Australia 8.88	4.83	13.71	81.01 [3]	0.53 [17]
16	France 1.52	Finland 4.96	Denmark 7.81	5.44	13.25	79.09 [14]	0.44 [7]
15	Denmark 1.33	Sweden 4.52	Canada 7.70	4.88	12.59	79.89 [10]	0.52 [16]
14	Italy 1.30	Australia 4.36	Spain 7.61	5.57	13.18	80.33 [7]	0.41 [3]
13	Canada 1.23	France 4.31	Austria 7.61	5.50	13.11	79.28 [13]	0.44 [8]
12	Portugal 1.19	Belgium 4.25	England and Wales 7.40	5.35	12.75	79.54 [11]	0.48 [14]
11	Australia 1.18	Canada 4.13	Italy 7.26	5.93	13.20	80.45 [6]	0.40 [2]
10	Ireland 1.15	Switzerland 4.03	The Netherlands 7.00	5.75	12.74	80.07 [9]	0.45 [9]
9	Sweden 1.14	Norway 4.00	Ireland 6.96	4.89	11.85	80.10 [8]	0.50 [15]
8	Japan 1.14	Portugal 3.90	Finland 6.66	5.29	11.95	78.72 [16]	0.44 [6]
7	The Netherlands 1.12	England and Wales 3.84	Japan 6.45	6.04	12.49	81.11 [2]	0.37 [1]
6	Belgium 1.08	Denmark 3.82	Belgium 6.32	5.07	11.40	79.00 [15]	0.46 [10]
5	Switzerland 1.07	Austria 3.72	France 6.10	5.27	11.36	79.45 [12]	0.47 [13]
4	Norway 1.03	The Netherlands 3.61	Sweden 6.01	5.69	11.70	80.73 [5]	0.44 [5]
3	Spain 0.98	Spain 3.33	Norway 5.80	5.13	10.92	80.92 [4]	0.46 [11]
2	Austria 0.89	Japan 3.13	Switzerland 5.42	5.57	10.99	81.37 [1]	0.46 [12]
1	Finland 0.89	Italy 3.06	Portugal 5.09	5.80	10.89	78.40 [17]	0.42 [4]

*Note*: Comparison with indicators of life expectancy at birth and coefficient of variation (standard deviation divided by mean) of the age-at-death distribution.

## Discussion

We exposed in this article one (among many) possible characterization of the distribution of age at death across populations with a focus on the lower and upper tails of the distribution. Such descriptive analyses might provide summary indicators of within country health disparities toward understanding how the concentrations of health might differ across better- and worse-off individuals. Some major advantages of this particular characterization of lifespan disparity (further described below) are that it is simple, intuitive and directly emulates commonly used income inequality measures.^21^ Our metrics, specifically the gap measures in age (δ) and share (Δ) across the 10th and 90th percentiles of the age-at-death distribution, additionally may help summarize national performances in health equity vis-à-vis stressing the position of the worse-off. The evolution in the age-at-death distribution over time can also point to the historical mortality trends experienced by countries (see selected illustrations in figure 1 and Supplementary figure SA6). For example, we can observe rapid declines in share gaps Δ during the accelerated mortality reduction progress post-World War II in Japan, while the gains were more steady in the USA (Supplementary figure SA6).

Overall, we observed great heterogeneity across countries and time, specifically for those percentile indicators pertaining to the bottom tail of the distribution ($a_{\leq 0.10}$); while, comparatively, we saw relatively more stability for the top tail of the distribution ($a_{\geq 0.90}$). This observation is largely due to substantial decreases in mortality among young age groups, notably associated with major reductions in communicable disease mortality that occurred during the first part of the 20th century (see $a_{0.10}$ and $a_{0.05}$ on figure 1).^13^^,^^14^^,^^16^^,^^25^^,^^26^ We highlight the great variety in the lower tails of the age-at-death distributions (large changes in $a_{0.01}$, $a_{0.05}$ and $a_{0.10}$) and how the shape of these tails may help pointing to the worse-off (the individuals with the smallest amount of longevity) as one possible feature of national health equity performance. Moreover, similar to economic studies where shares of income distributions across percentiles are scrutinized (Supplementary figure SA7),^21^ we stress the differences in shares of longevity (with shares F and share gaps Δ). In particular, we could point to the usefulness of the indicator $\Delta_{0.10}$, capturing the difference in longevity shares between the 10th and 90th percentiles of the age-at-death distribution, and providing information complementary to the traditional dispersion metrics (e.g. coefficients of variation; table 1). Lastly, while income inequalities seem to have either widened (e.g. in the USA and UK) or been maintained for the last 40 years,^21^ it appears difficult to draw consistent associations for the evolution of our longevity shares (Supplementary figures SA7 and SA8).

Our analysis presents a number of important limitations. First and foremost, we have only pursued a descriptive analysis. In doing so, we build on a large published literature (e.g. see: Keyfitz and Casewell,^8^ Wilmoth and Horiuchi,^9^ Shkolnikov *et al*.,^10^ Shkolnikov *et al*.,^11^ van Raalte *et al*.,^12^ Aburto *et al*.,^13^ Vaupel *et al*.,^14^ Németh *et al*.,^16^ Jamison *et al*.,^15^ Edwards and Tuljapurkar,^27^ Engelman *et al*.,^28^ Permanyer and Scholl,^29^ Mejía and Tuljapurkar,^30^ Kannisto,^31^ Zuo *et al*.^32^ and Myers and Menton^33^). Yet, we do not attempt to offer any underlying mechanisms to explain the cross-country differences observed, as notable analyses of the social determinants of health can do.^4^^,^^34^ Second, there exist a few limitations with the data at hand. Some countries (e.g. Sweden since 1751) as opposed to others (e.g. Ireland since 1950) have either more or less available data, and the sample of the countries studied was limited by the country-years available within the HMD. For instance, we could not include a number of largely populated countries with emerging economies (like Brazil, China, India and Nigeria) and were restricted to high-income countries.^22^ Furthermore, within the HMD, there is much quality heterogeneity across the country-years available; some data, particularly for old time periods and older ages (i.e. relevant for $a_{0.90}$ and $a_{0.95}$, $a_{0.99}$ estimates) can be modeled.^35–37^ Third, some of our computations used a number of simplifications. For example, extracting the 1st and 99th percentiles was difficult due to small numbers of deaths reported at these percentiles. This leads to difficulties in estimating stable quantities for $a_{0.01}$, $a_{0.99}$, $\delta_{0.01}$ and $\Delta_{0.01}$, which should then be interpreted with great caution. This is one of the reasons that prompted us to focus on the $\delta_{\left\{0.05;\;0.10\right\}}$ and $\Delta_{\left\{0.05;\;0.10\right\}}$ quantities. Fourth, our approach drawing from age-at-death distributions is only one simple way of characterizing the dispersion in those distributions. Indeed, many alternative measures have been used including interquartile ranges, coefficients of variations, in addition to the commonly implemented Gini and concentration indices, etc.^38^ There is a fairly large literature that has already used various cutoffs of age-at-death distributions to capture changes in lifespan disparities over time. Importantly, Kannisto^31^ used ‘C-indices’ [minimum age ranges containing a given share of deaths; e.g. C90 would contain 90% of deaths and be comparable (yet distinct) to our $\delta_{0.05}$ indicator] to quantify the evolution of mortality compression. Zuo *et al*.^32^ also used percentile-based approaches to study old-age deaths in high-income countries. More broadly, there exist numerous studies on mortality concentration and compression using similar indicators based off age-at-death distributions.^27–30^^,^^33^ Yet, to our knowledge, the use of percentile-based approaches for the systematic comparison of country performance in health equity has remained small.

In summary, we hope to point to the easiness and relevance of reporting on the extreme tails of age-at-death distributions as a way to characterize the situations of the better- and worse-off individuals across nations, similarly to the routine depictions of income distributions. Yet, with this descriptive analysis, we only offer a set of preliminary investigations with the intent to merely lay the ground for future sophisticated and systematic cross-country analyses and discussions.

## Supplementary data

Supplementary data are available at *EURPUB* online.

## Supplementary Material

ckac134_Supplementary_Data
