# Supplementary material for: Top and bottom longevity of nations: a retrospective analysis of the age-at-death distribution across 18 OECD countries
Source: Eur J Public Health. 2022 Oct 14;33(1):114–20. doi: 10.1093/eurpub/ckac134 (PMC10132764; doi:10.1093/eurpub/ckac134)
Supplement: ckac134_Supplementary_Data [file ckac134_supplementary_data.pdf]

Supplementary webappendix

Table A1. Summary characteristics of the data sourced from the Human Mortality Database.

Country	Time-period	Number of years
Australia	1921-2018	98
Austria	1947-2019	73
Belgium	1900-2020*	121
Canada	1921-2019	99
Denmark	1900-2020	121
England & Wales	1900-2018	119
Finland	1900-2020	121
France	1900-2018	119
Ireland	1950-2017	68
Italy	1900-2018	119
Japan	1947-2019	73
Netherlands	1900-2019	120
Norway	1900-2020	121
Portugal	1940-2020	81
Spain	1908-2018	111
Sweden	1900-2020	121
Switzerland	1900-2020	121
United States of America	1933-2019	87

*No data available for 1914-1918.

Figure A1. Distance (gap) across ages at death (in years) between top and bottom percentiles of the age-at-death distribution: $\delta_{0.10} = a_{0.90} - a_{0.10}$, $\delta_{0.05} = a_{0.95} - a_{0.05}$, and $\delta_{0.01} = a_{0.99} - a_{0.01}$. Denmark, England & Wales, France, Italy, the Netherlands, and Sweden; females and males; 1900-2020.

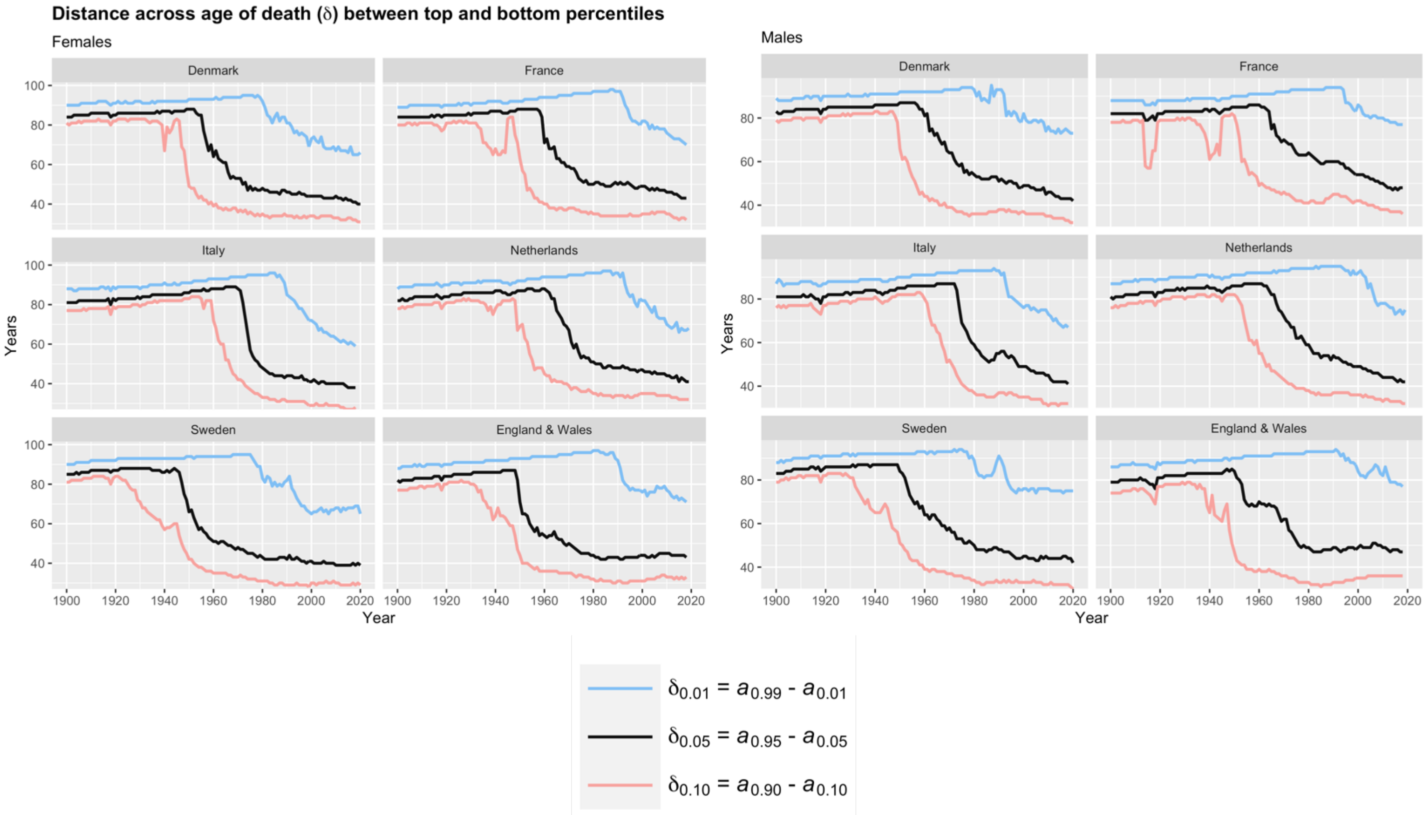

Figure A2. Gap in the fraction of health (longevity) between top and bottom percentiles of the age-at-death distribution: $\Delta_{0.10} = F_{0.90} - F_{0.10}$, $\Delta_{0.05} = F_{0.95} - F_{0.05}$, and $\Delta_{0.01} = F_{0.99} - F_{0.01}$. Denmark, England & Wales, France, Italy, the Netherlands, and Sweden; females and males; 1900-2020.

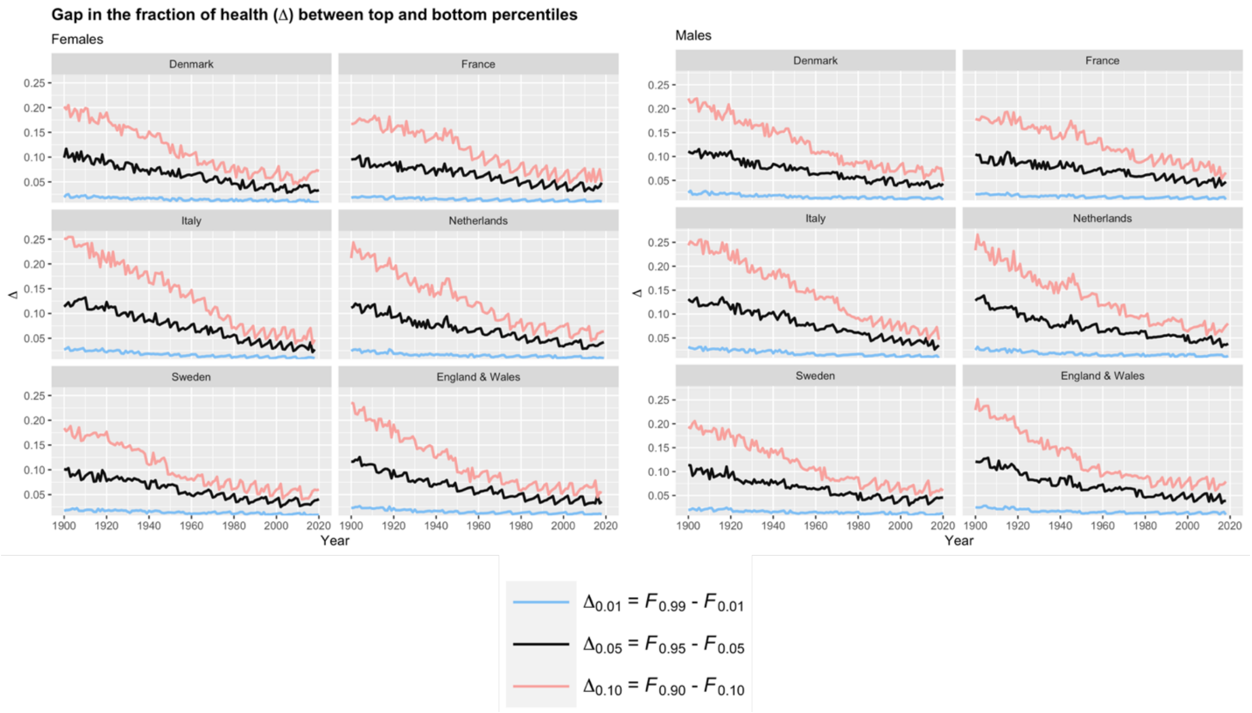

Figure A3. First calendar year in which the percentile of the age-at-death distribution became greater than 0 years: $a_{0.10} > 0$ (10); $a_{0.05} > 0$ (5), and $a_{0.01} > 0$ (10); 18 countries; females and males.

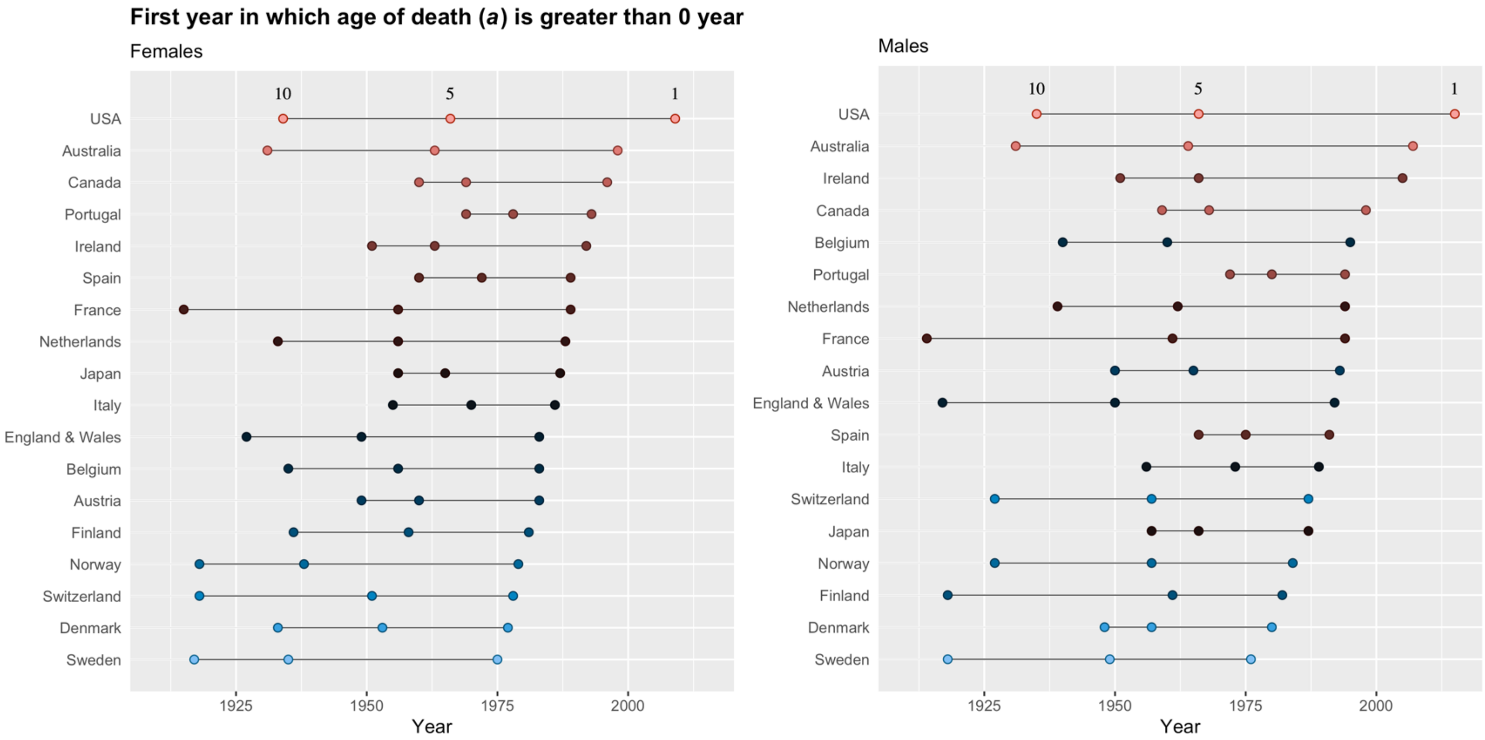

Figure A4. Variation (including median, interquartile range) in the share of longevity, that is $F_{0.01}$, $F_{0.05}$, $F_{0.10}$, $F_{0.90}$, $F_{0.95}$, and $F_{0.99}$ of the percentiles of the age-at-death distribution. All country-years since 2000.

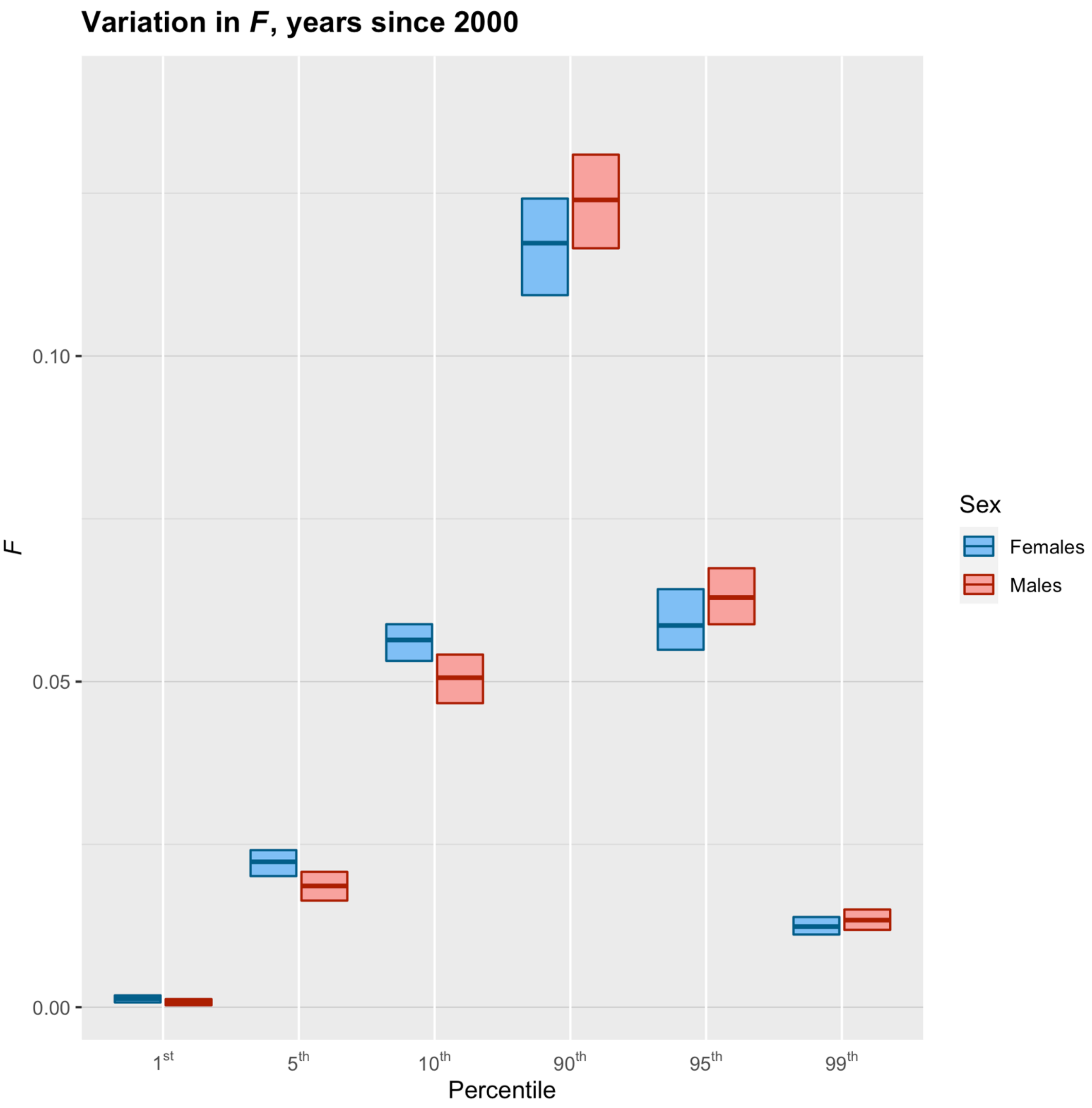

Figure A5. Variation (including median, interquartile range) in the gap in the fraction of longevity between top and bottom percentiles of the age-at-death distribution: $\Delta_{0.10}= F_{0.90} - F_{0.10}$ (10), $\Delta_{0.05}= F_{0.95} - F_{0.05}$ (5), and $\Delta_{0.01}= F_{0.99} - F_{0.01}$ (1); all country-years since 2000.

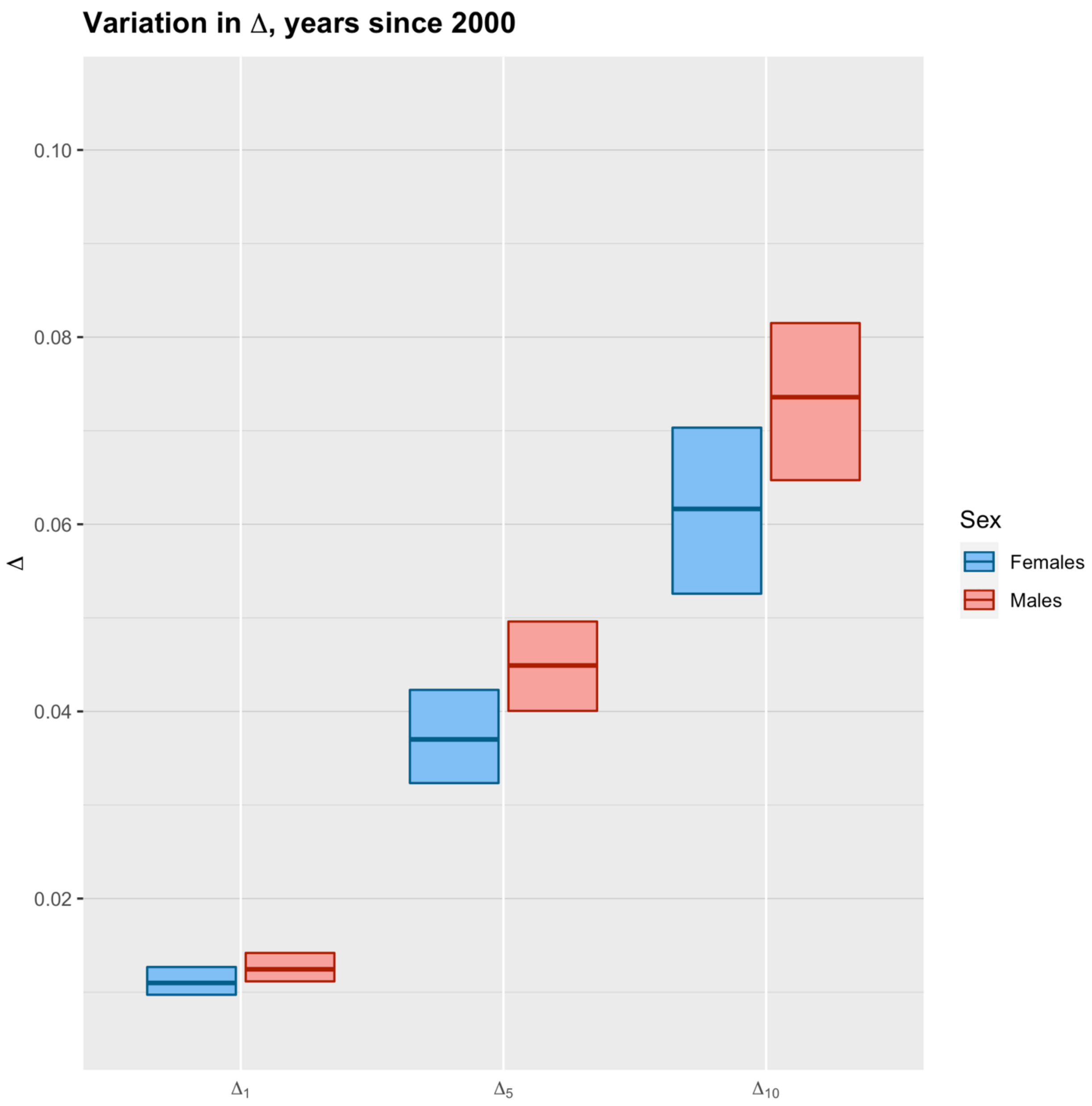

Figure A6. Gap in the fraction of health (longevity) between top and bottom percentiles of the age-at-death distribution: $\Delta_{0.10} = F_{0.90} - F_{0.10}$, $\Delta_{0.05} = F_{0.95} - F_{0.05}$, and $\Delta_{0.01} = F_{0.99} - F_{0.01}$. Japan and USA; females and males; 1947-2019.

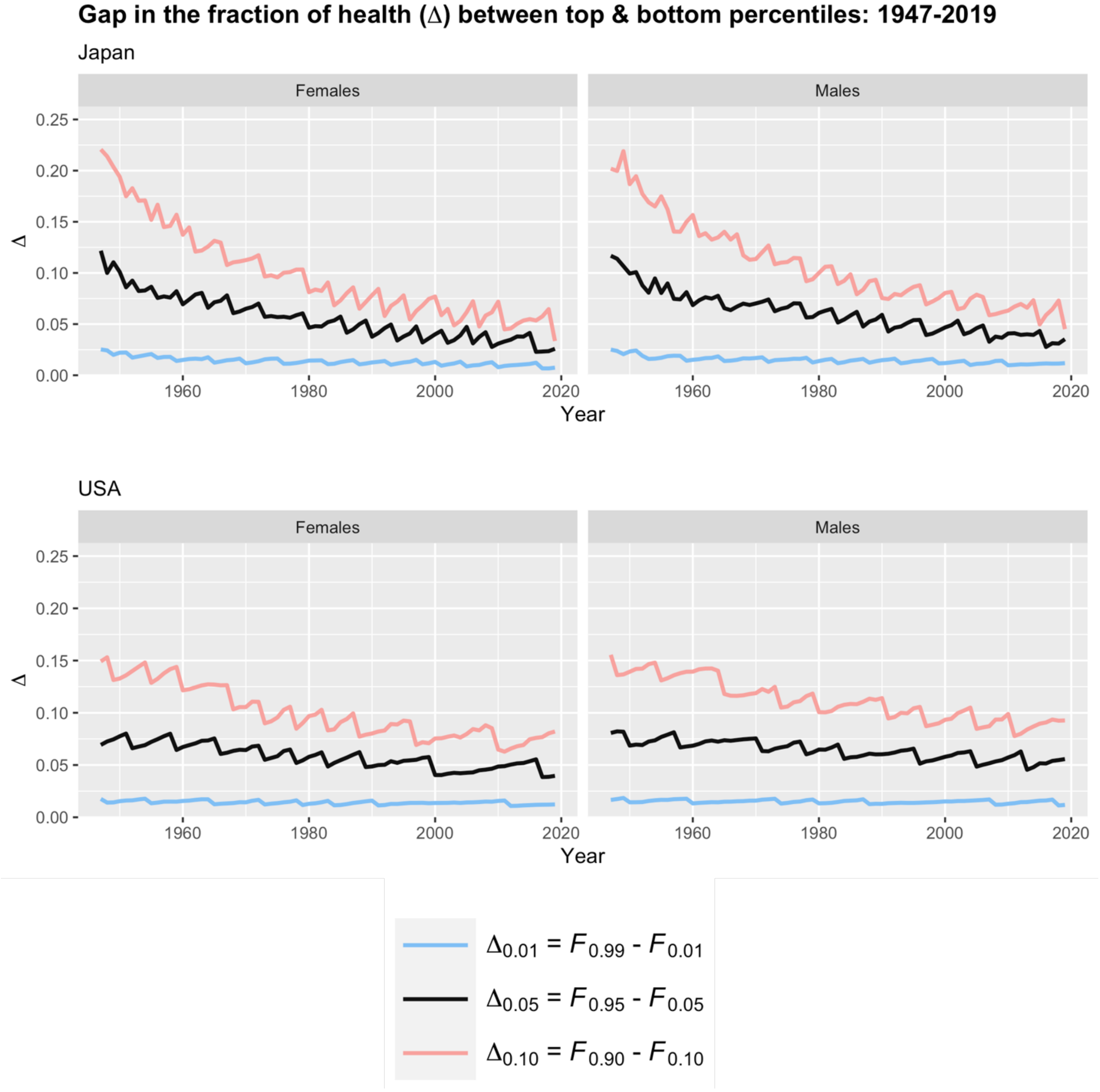

Figure A7. Share of total income by percentile of the income distribution, for the bottom 50%, top 10%, and top 1% of the income distribution; 18 countries, 1900 onwards.

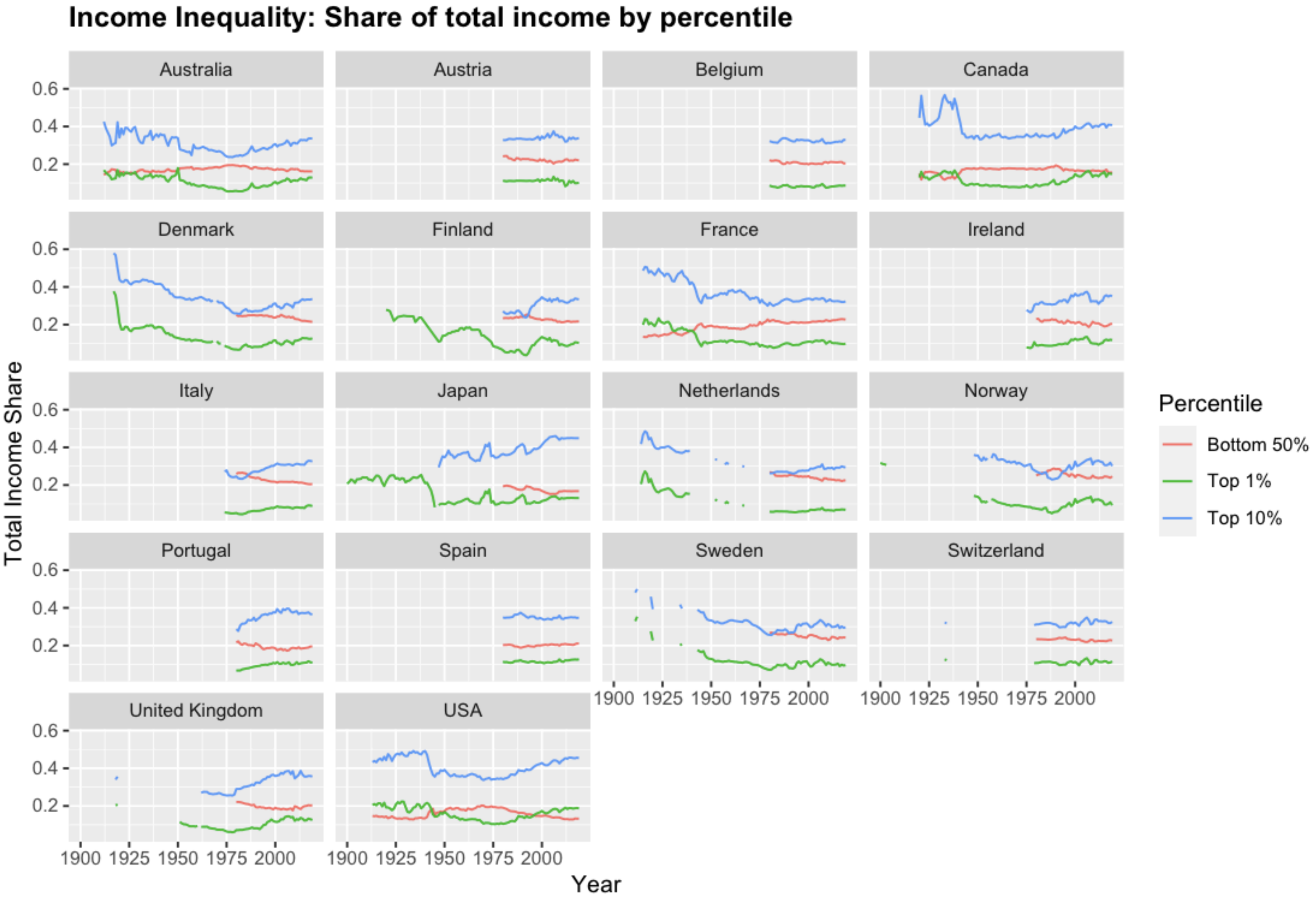

Source: Chancel et al. (2022).

Figure A8. Health (longevity) gap between the 10th and 90th percentiles of the age-at-death distribution ($\Delta_{0.10} = F_{0.90} - F_{0.10}$) (y-axis) and share of total income for the top 10% of the income distribution; 17 countries, 1900 onwards (x-axis).

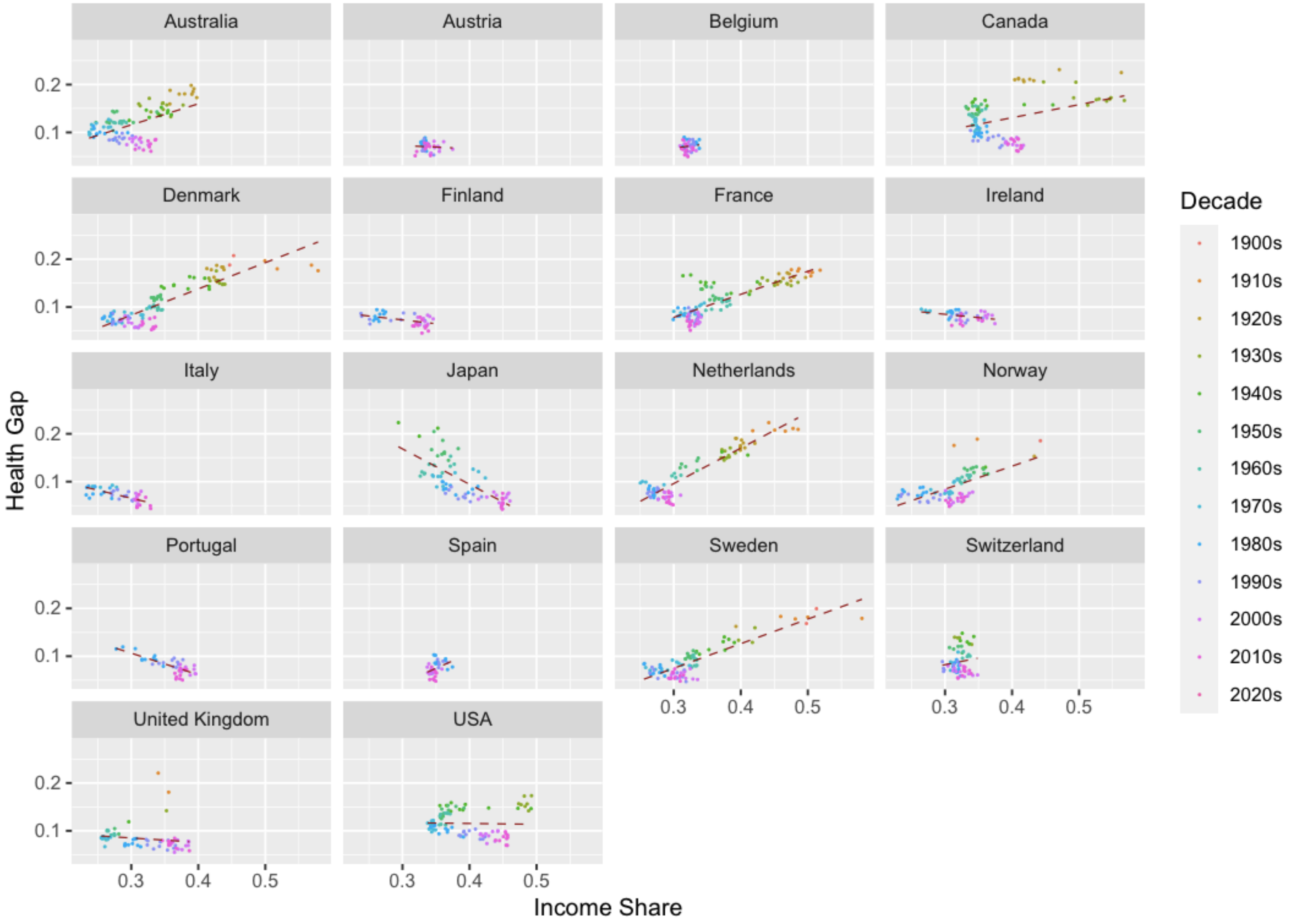

Source: Authors' calculations; Chancel et al. (2022).

References

Chancel L, Piketty T, Saez E, Zucman G. *World Inequality Report 2022*. World Inequality Lab.
